# Differential cellular FGF-2 upregulation in the rat facial nucleus following axotomy, functional electrical stimulation and corticosterone: a possible therapeutic target to Bell's palsy

**DOI:** 10.1186/1749-7221-5-16

**Published:** 2010-11-09

**Authors:** Karen F Coracini, Caio J Fernandes, Almir F Barbarini, César M Silva, Rodrigo T Scabello, Gabriela P Oliveira, Gerson Chadi

**Affiliations:** 1 Department of Neurology, University of São Paulo, Av. Dr. Arnaldo, 455 2nd floor, room 2119, São Paulo - 01246-903, Brazil

## Abstract

**Background:**

The etiology of Bell's palsy can vary but anterograde axonal degeneration may delay spontaneous functional recovery leading the necessity of therapeutic interventions. Corticotherapy and/or complementary rehabilitation interventions have been employed. Thus the natural history of the disease reports to a neurotrophic resistance of adult facial motoneurons leading a favorable evolution however the related molecular mechanisms that might be therapeutically addressed in the resistant cases are not known. Fibroblast growth factor-2 (FGF-2) pathway signaling is a potential candidate for therapeutic development because its role on wound repair and autocrine/paracrine trophic mechanisms in the lesioned nervous system.

**Methods:**

Adult rats received unilateral facial nerve crush, transection with amputation of nerve branches, or sham operation. Other group of unlesioned rats received a daily functional electrical stimulation in the levator labii superioris muscle (1 mA, 30 Hz, square wave) or systemic corticosterone (10 mgkg^-1^). Animals were sacrificed seven days later.

**Results:**

Crush and transection lesions promoted no changes in the number of neurons but increased the neurofilament in the neuronal neuropil of axotomized facial nuclei. Axotomy also elevated the number of GFAP astrocytes (143% after crush; 277% after transection) and nuclear FGF-2 (57% after transection) in astrocytes (confirmed by two-color immunoperoxidase) in the ipsilateral facial nucleus. Image analysis reveled that a seven days functional electrical stimulation or corticosterone led to elevations of FGF-2 in the cytoplasm of neurons and in the nucleus of reactive astrocytes, respectively, without astrocytic reaction.

**Conclusion:**

FGF-2 may exert paracrine/autocrine trophic actions in the facial nucleus and may be relevant as a therapeutic target to Bell's palsy.

## Background

It is important the knowledge on the molecules involved in the trophic mechanisms of motoneurons in order to develop therapeutic targets to peripheral nerve disorders which are the case of facial nerve in the Bell's palsy. The disease usually does not last long and undergoes spontaneous recovery in many cases but sometimes therapeutic interventions are necessary to reduce the symptoms or when amelioration is not achieved.

In the disorder, the compromised facial nerve swells up and presses against its trajectory inside the temporal bone, being squashed and functionally/anatomically impaired [1]. Around one in five people will suffer long lasting symptoms. In patients presenting incomplete facial palsy and probably bearing only functional impairments, the prognosis for recovery is very good and treatment may be unnecessary. On the other hand, patients presenting complete paralysis, marked by an inability to close the eyes and mouth on the involved side, that received early treatment might show a favorable response by 3-12 months [2]. This indicated that injured facial neurons can be rescued and might undergo regeneration, a process that takes time considering the distance to facial muscle targets. However, some cases are resistant to current proposed treatments which are mainly based on antiinflammatory drugs and local neuromuscular manipulations [3].

Different from peripheral sensory neurons that seem to be less resistant to axotomy probably because of a high dependence of trophic support from their innervation targets, the majority of adult peripheral motoneurons survive after an injury of their fibers. Motoneuron trophism is probably a result of autocrine/paracrine mechanisms which take place at cell perykaria that are able to the rescue axotomized cells. Moreover, the protection of neuronal cell bodies from degeneration is essential for axonal regeneration and similar cell signaling might be involved in both events [4].

Basic fibroblast growth factor (FGF-2, bFGF) is a mitogenic protein capable of acting on multiple cell types such as neurons and glial cells [5]. FGF-2 protein and messenger RNA (mRNA) have been found in the cytoplasm of neurons and in the nuclei of astrocytes of many brain regions [5-8]. FGF-2 plays a role in the neuronal development in prenatal life and also influence survival and plasticity of mature central nervous system (CNS) neurons [9,10]. Furthermore, paracrine actions of the astroglial FGF-2 have been described following postnatal CNS lesions [11,12].

Lesions to the CNS have been described to induce a strong expression of FGF-2 mRNA and protein in activated astroglial cells in the area of the injury [11-14]. Although an increasing number of studies have pointed out the role of FGF-2 following cellular lesion, few works have attempted to investigate cellular regulation of FGF-2 in response to axotomy of the peripheral motoneurons. It is likely that the ability of adult peripheral motoneurons to survive after axotomy is probably due to multiple cellular sources of trophic support [15-18]. This feature must be better interpreted in order to achieve effective therapeutic targets leading to benefits for those patients with impaired functional recovery after Bell's palsy.

The present work analyzed the neuronal and glial responses as well as cellular FGF-2 regulation in the facial nucleus following a cervical crush or transection, with amputation of nerve branches, of facial nerve of the adult Wistar rat. We have also examined the effects of systemic corticosterone and functional electrical stimulation applied in a facial muscle on FGF-2 expression in non axotomized facial nuclei.

## Methods

### Animals and experimental procedures

Specific pathogen-free adult male Wistar rats (University of São Paulo, Medical Scholl) of 250 g body weight (b.w.) were used in the experiments. The animals were kept under standardized lighting conditions (light on at 7:00 h and off at 19:00 h), at a constant temperature of 23°C and with free access to food pellets and tap water. The study was conducted according protocols approved by the Animal Care and Use of Ethic Committee at the University of São Paulo and in accordance with the Guide for Care and Use of Laboratory Animals adopted by the National Institutes of Health.

### Facial nerve injury

In the first set of experiments, rats (n = 18) were submitted to a sham-operation, a crush or a transection of the facial nerve as described. Briefly, under sodium pentobarbital (45 mgkg^-1^, Cristalia, São Paulo, Brazil) anesthesia, the rat facial skin of the right side was opened near the ear and the facial nerve of that side was isolated. Following, the facial nerves were crushed (n = 6) twice with a pair of Dumont #5 forceps for 30 sec, 3 mm from the stylomastoid foramen or completely transected (n = 6) with delicate tweezers being the distal and proximal nerve stumps inverted and tied. In the sham-operated animals (n = 6) the facial nerves were exposed and isolated in an identical manner but they were not axotomyzed. Animals were sacrificed 7 days after the surgery and their brain processed for immunohistochemistry.

### Systemic drug injection and functional electrical stimulation

In a second set of experiments employing unlesioned rats, effects of systemic corticosterone injection or local functional electrical stimulation were evaluated on non axotomized facial nuclei. In a group of rats, animals received systemic daily injections of corticosterone (10 mg × kg^-1 ^b.w., ip., n = 6) or solvent (n = 6) for seven days. Corticosterone (Sigma, USA) was suspended in deionized water solution containing carboxymethylcellulose natrium salt (0.25% w/v; Sigma) and polyoxyethylene sorbital mono-oleate (tween 80, 0.2% v/v; Sigma). All injections were made in the afternoon to mimic the endogenous peak of corticosterone secretions and the solvent was given in the same volume and in the same time as the corticosterone injections. This high dose of corticosterone was chosen, since it is a standard dose used to mimic the stress level of corticosterone [19].

Other group of rats with unlesioned facial nerve was submitted to a functional electrical stimulation according to protocols of Miles [20], Pilyavskii [21] and of Blum [22] adapted for facial muscles by our group. Briefly, a thread electrode for stimulation (1.0 cm long/0.7 mm thickness) made of stainless steel fixed in a silicone-isolated copper thread was connected to an electrical stimulator. Twenty-four hours prior first stimulation, animals were anaesthetized (a combination of S (+)-ketamin cloridrate, 62.5 mg × kg^-1 ^and xilazine cloridrate, 10 mg × kg^-1^, respectively from Cristalia and Vetbrands, São Paulo, Brazil) and submitted to a surgical procedure in order to expose the right side of levator labii superioris muscle and to perform local implantation of a thread electrode which was fixed by means of a surgical 10-0 mononylon thread. After a short trajectory through the subcutaneous layer, the silicone-isolated copper thread was exteriorized through a small aperture in the dorsal surface of the rat neck. The tip of that exteriorized thread was daily connected to the electrical stimulator only during the period of stimulation sections. Furthermore, a second electrode was fixed in the skin/subcutaneous layer to ground the stimulation. The procedure was validated by examining the muscle response after stimulation. Animals not showing visible contractions or vibrissal movements, or requiring currents higher that 1 mA were discarded. Twenty-four hours later, awake and free moving animals were submitted to the electrical stimulation protocol by means of a 4-channels-electrical stimulation (Vif FES 4, Quark, Brazil). The stimuli consisted of a 1 mA current, 30 Hz frequency with a square wave (5 sec on/10 sec off), which was applied daily, for 30 min in the beginning of the morning. Control rats were submitted to electrode surgical implantations, daily connected to stimulator without receiving the electrical stimulation.

Animals of the second set of experiments were also sacrificed 7 days after the beginning of the procedures and their brain processed for immunohistochemistry.

### Tissue processing

After the experimental procedures described above, rats were deeply anaesthetized with sodium pentobarbital 10% (420 mg/kg/b.w., i.p.) and euthanized by a perfusion through a cannula inserted in the ascending aorta with 50 ml of isotonic saline at room temperature followed by 350 ml of fixation fluid (4°C) during 6 min as described previously [23,24]. The fixative consisted of 4% (w/v) paraformaldehyde and 0.2% (v/v) picric acid in 0.1M phosphate buffer (pH 6.9). The brains were dissected out and kept in the fixative solution for 90 min. The fixed brains were washed in 10% sucrose dissolved in 0.1M phosphate buffered saline (PBS pH 7.4) for 2 days, frozen in ice-cold isopentane and stored at -70°C. Coronal brain sections (14 μm thick) were made through the facial nucleus from bregma level -11.60 mm to -10.3 mm, according to the atlas of Paxinos & Watson [25], using a Leica cryostat (CM 3000, Germany). Sections were sampled systematically and six series in a rostrocaudal order including every sixth section were used for immunohistochemistry. The analyses were performed in the facial nuclei bilaterally.

The series of thaw-mounted sections were incubated overnight at 4°C in a humidified chamber with one of the following antisera: a rabbit polyclonal FGF-2 antiserum against the bovine FGF-2 [26] (diluted 1:800), a rabbit polyclonal antiserum against the glial fibrillary acidic protein (GFAP, 1:1500, Dakopats, Danmark) or a mouse monoclonal antiserum against the neurofilament (NF, only in the experiments of facial nerve injury) of molecular weight 200 kDa (1:1000) (Sigma, USA). The antibodies were diluted in PBS containing 0.3% Triton X-100 (Sigma) and 0.5% bovine serum albumin (Sigma). The detection of the antibodies was achieved by the indirect immunoperoxidase method using the avidin-biotin peroxidase (ABC) technique as previously described [27-29]. After washing in PBS (3 × 10 min), the sections were incubated with a biotinylated goat anti-rabbit or biotinylated horse anti-mouse antibodies (both diluted 1:200, Vector, USA) for one hour. In a third step, sections were washed in PBS and incubated with avidin-biotin peroxidase complex (both diluted 1:100, Vectastain, Vector) during 45 min. The staining was performed using 0.03% of 3,3'-diaminobenzidine tetrahydrochloride (DAB, Sigma) as a chromogen and 0.05% (v/v) of H_2_O_2 _(Sigma) during 6-8 min, which gave a brownish color to the immunoreaction. Duplicate series of NF and GFAP immunoreactive sections from the facial nerve injury were stained by cresyl violet (CV) for *interalia *visualization of Nissl substance. For standardization of the immunohistochemical procedure we have used a dilution of the primary antibody and a DAB concentration far from saturation and an incubation time adjusted so that the darkest elements in the brain sections were below saturation. The FGF-2 antiserum is a well characterized polyclonal antiserum raised against the *n *terminal (residues 1-24) of the synthetic peptide of bovine FGF-2 (1-146) [26]. This antiserum does not recognize acidic FGF (cross reactivity less than 1%) [11]. As control, sections were incubated overnight at 4°C with the FGF-2 antiserum (diluted 1:800) pre-incubated with human recombinant FGF-2 (50 μg/ml, for 24 h at 4°C). For a further analysis of the immunostaining specificity, sections were also incubated with the solvent of the primary or secondary antibody solutions as well as the solvent of the avidin-biotin solution and processed simultaneously in the experimental sections.

The two-color immunoperoxidase method was employed in a series of sections for a simultaneous detection of the FGF-2 and GFAP immunoreactivities. The FGF-2 immunoreactivity was firstly demonstrated as described above. Following the DAB reaction, the sections were rinsed several times in PBS and were incubated during 48 h in a humidified chamber with the rabbit polyclonal antiserum against GFAP described above (1:500). After several rinses in PBS, the sections were incubated with biotinylated goat anti-rabbit immunoglobulins (1:200, Vector) for 1 h at room temperature and with an avidin and biotin peroxidase solution (both diluted, 1:100; Vectastain, Vector) for 45 min at room temperature. The staining was performed using 4-chloro naphthol 0.05% (Sigma) as a chromogen and 0.05% (v/v) H_2_O_2 _(Sigma) during 10 min. This procedure gave a brownish color to the FGF-2 immunoreactivity and a bluish a color to the GFAP immunoreaction. The immunoreactivities were also analyzed qualitatively and photographed in an Olympus AX70 photomicroscope (USA).

### Quantitative analysis

#### Cell Counting

The NF+CV neuronal profiles, GFAP+CV astroglial profiles and the glial FGF-2 immunoreactive profiles from the facial nerve injury experiment were counted under *camera lucida *microscopy at 16× magnification mounted in a Zeiss microscope (Germany). An area of 116.39 μm2 was sampled in the central region of the right side (lesioned side) and the left side (control side) of the facial nucleus and the profiles were counted. The cytoplasmatic and nuclear localization of the FGF-2 immunoreactivity [9] were taken into account in the discrimination of the neuronal and glial FGF-2 cell profiles. In order to minimize individual variability, the data were presented and evaluated statistically as the quotient of ipsi *vs *contralateral sides.

#### Semiquantitative microdensitometric image analysis

FGF-2 immunoreactivity in sections from experiments of systemic corticosterone injection and facial functional electrical stimulation of unlesioned facial nerve rats was submitted to semi quantitative image analysis measurements. We have not performed a cell counting in the unlesioned animals because the qualitative evaluations showed a major change in the intensity of FGF-2 immunoreactivity per cell profiles and not in the number of profiles. To maximize the intensities of the FGF-2 immunoreactivity on neuronal and glial profiles, this analysis was performed on sections of rat brains from the rostro-caudal levels described above [29,30]. Fields of measurements were sampled in the central regions of the facial nuclei bilaterally. The procedures using a Kontron-Zeiss KS400 image analyzer (Germany) have been described previously [9,30-32]. Briefly, a television camera acquired images from the microscope (40× objectives). After shading correction, a discrimination procedure was performed as follows: the mean gray value (MGV) and S.E.M. of white matter was measured in an area of the medulla oblongata devoid of specific labeling (background, bg). Gray values darker than bgMGV-3 S.E.M. were considered specific labeling. The specific (sp) MGV was then defined as the difference between the bgMGV value and the MGV of the discriminated profiles. The size of the sampled field was 2.56 × 10^-2^mm^2^. This parameter reflects the immunoreactive intensities in the discriminated profiles (spMGV) and indicates, semiquantitatively, the amount per profile of the measured immunoreactivity. The area of discriminated profiles within the sample fields was also registered and reflects the amount of profiles processing the immunoreactive product. The glass value was kept constant at 200 MGV. The procedure was repeated for each section to correct every specific labeling measurement for background. Moreover, DAB and H_2_O_2 _were used in optimal concentrations and FGF-2 antibody dilution was far from saturation. Under these conditions, the steric hindrance of peroxidase complex does not appear to disturb the linear relationship between antigen content and staining intensity. However, in the absence of a standard curve, the relationship between antigen content and staining intensity is unknown, and the results must be considered as semiquantitative evaluations of the amount of antigen present in the sampled field. Thus, spMGV only gives semiquantitative evaluations of the intensity of FGF-2 immunoreactivity [33]. In the corticosterone experiments, the data represent mean of the bilateral measurements and in the functional electrical stimulation experiments, the data represent the quotient of ipsilateral *vs *contralateral sides.

The statistical analysis was performed using the non-parametric Mann-Whitney *U*-test [34]. The number of each animal represents the Mean ± S.E.M. obtained in each side of the facial nuclei of the sampled sections.

## Results

### Axotomy of facial nerve

**Figure 1:**
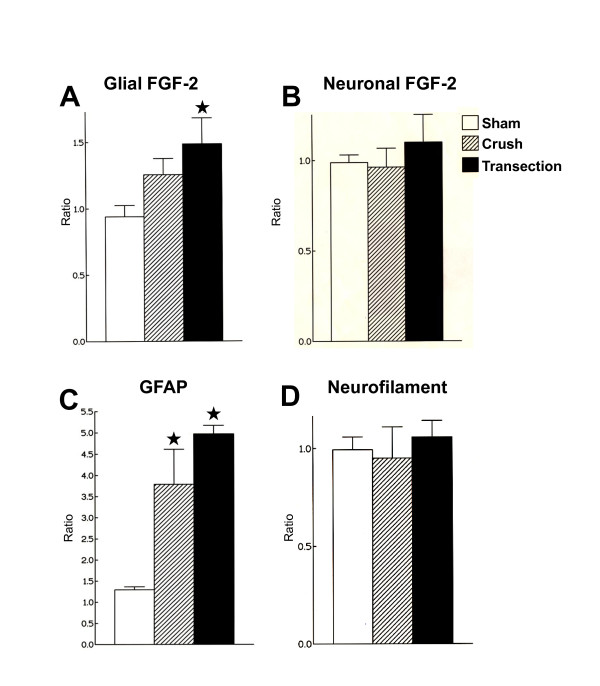
**Effects of the unilateral crush or transection of facial nerve on FGF-2 immunoreactive data**. Ratio number of fibroblast growth factor-2 (FGF-2) immunoreactive glial (**A**) and neuronal (**B**) profiles, of glial fibrillary acidic protein (GFAP) immunoreactive astrocytic profiles (**C**), neurofilament plus cresyl violet immunoreactive neuronal profiles (**D**) of the facial nucleus of the rats. The vertical axis represents the ratio of the number of immunoreactive profiles in the ipsilateral versus contralateral nucleus. Animals were studied 7 days after injury (means ± S.E.M., n = 6). *p < 0.05 according to the non-parametric Mann-Whitney *U *test.

**Figure 2:**
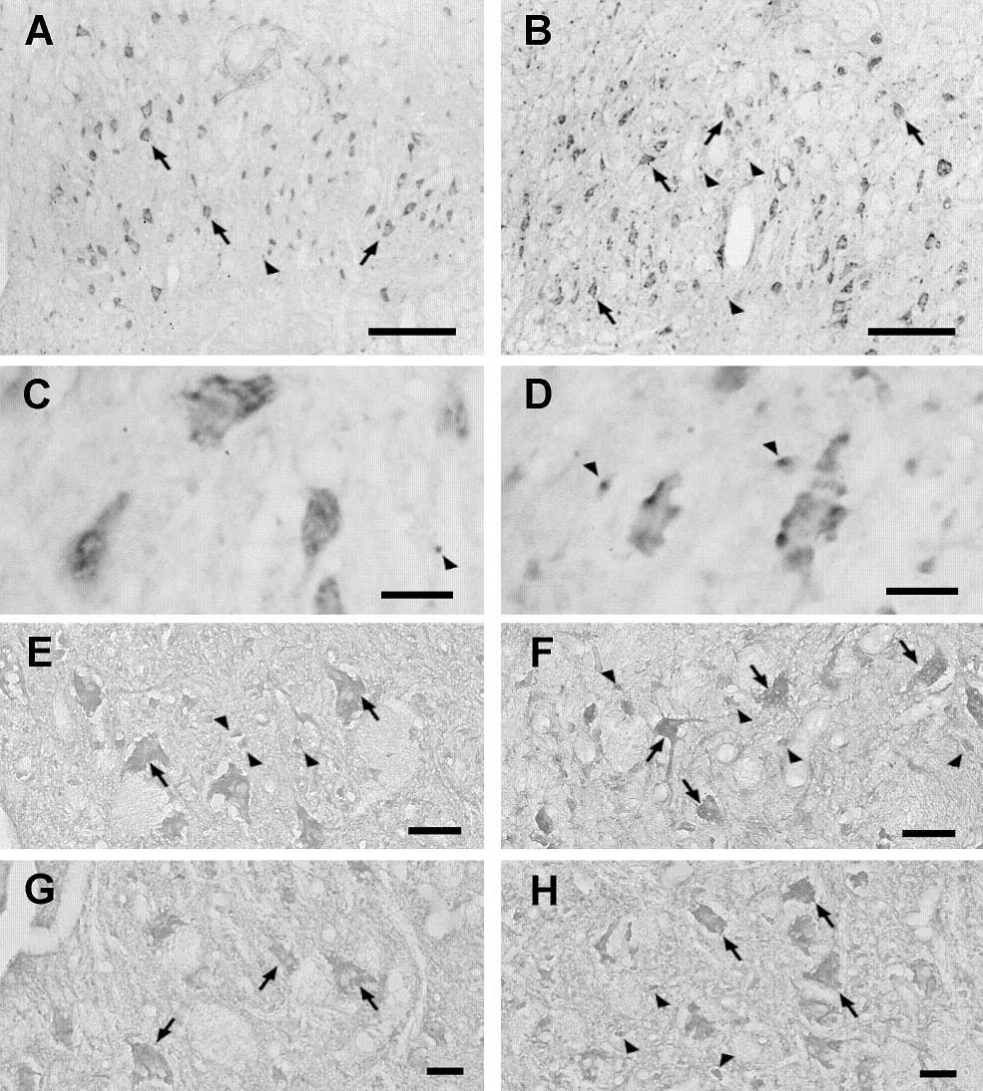
**Microphotographs showing fibroblast growth factor (FGF-2) immunoreactivity in coronal sections of rat facial nucleus**. Animals were submitted to the following procedures and sacrificed 7 days later: a transection of the facial nerve (with amputation of the nerve stumps) **(B**, **D) **or a sham operation **(A**, **C)**; a 7-days systemic treatment of corticosterone (daily ip. injection of 10 mg × kg^-1^, corticosterone) (**E**) or solvent (**F**); a 7-days unilateral functional electrical stimulation of the levator labii superioris muscle after a local implantation of a mononylon thread electrode (1 mA current, 30 Hz frequency square wave) (**G**) or without current as control (**H**). The facial nerve was not lesioned in the corticosterone and electrical stimulation experiments. The figures **C **and **D **represent higher magnification of areas inside the nuclei showed in figure **A **and **B**, respectively. The FGF-2 immunoreactivity is seen in the cytoplasm of neurons (arrows) and in the nuclei of glial cells (arrowheads), respectively. It is observed that the transection of the facial nerve and also systemic corticosterone increased the FGF-2 immunoreactivity in the nuclei of glial cells in facial nuclei ipsilateral to the injury and bilaterally after drug injection. The functional electrical stimulation of the levator labii superioris led to increase of FGF-2 mainly in the cytoplasm of neurons of facial nucleus ipsilateraly. Bars = 50 μm (**A**, **B**), 25 μm (**C-H**).

Increases in the number of the FGF-2 immunoreactive nuclear glial profiles were found in the ipsilateral facial nuclei seven days after both methods of axotomy, however significance was reached after nerve transection with amputation of nerve stumps (57.97%, Figure 1A, illustrated in Figure 2A-D). Moreover, no statistical differences were obtained between crush and transection regarding the number of FGF-2 immunoreactive profiles (Figure 1A). Despites facial nerve crush and transection have promoted no changes in the number of the FGF-2 immunoreactivity of neuronal profiles in the lesioned side (Figure 1B), the intensity of the FGF-2 immunoreactivity increased slightly in the cytoplasm of neuronal profiles seven days after the axotomy as evaluated qualitatively by means of a direct microscopic analysis (Figure 2A-D).

**Figure 3:**
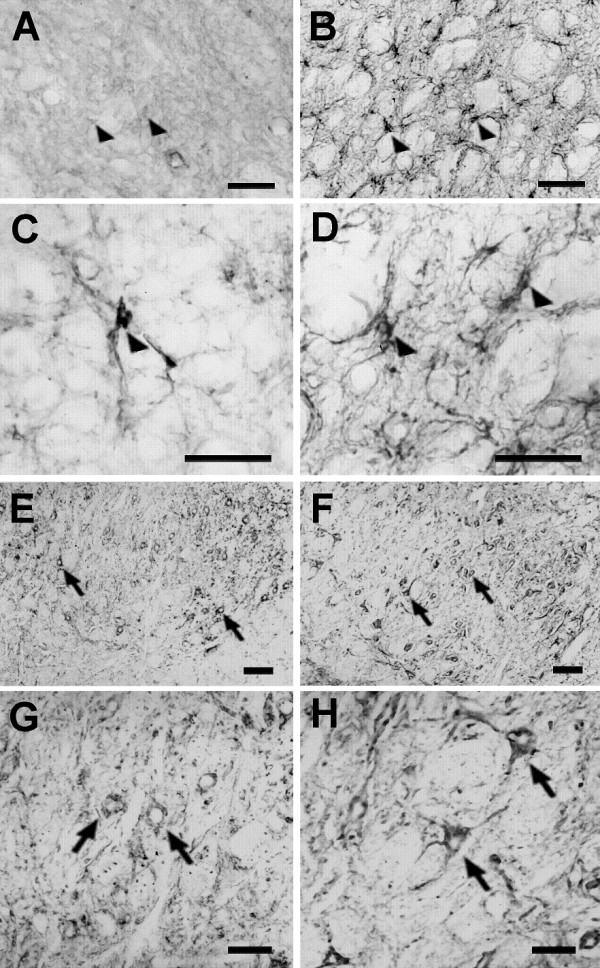
**Microphotographs showing rat facial nuclei submitted to immunohistochemistry of different markers**. Animals were submitted to the transection of the facial nerve (with amputation of the nerve stumps) **(B**, **D**, **F**, **H) **or submitted to a sham operation **(A**, **C**, **E**, **G)**, 7 days before the sacrifice. The figures **A-D **show glial fibrillary acidic protein (GFAP) immunoreactivity, figures **E-H **show neurofilament (NF) ones in coronal sections of the facial nucleus of rats. The figures **C**, **D **and **G**, **H **represent higher magnification of areas inside the nuclei showed in figure **A**, **B **and **E**, **F**, respectively. Arrowheads show GFAP immunoreactive astrocytes and arrows point to NF immunoreactive neurons. The GFAP immunoreactivity is increased in the cytoplasm and processes of astrocytes of the facial nucleus of the lesioned rats (**B**, **D**). Furthermore, NF immunoreactivity is increased in the cell body of neurons and neuropil of the facial nucleus of the lesioned rats (**F**, **H**). Bars = 100 μm **(E**, **F)**, 50 μm **(A**, **B**, **G**, **H)**, 25 μm (**C**, **D**).

The number of the GFAP immunoreactive glial profiles increased in the ipsilateral facial nuclei of the crushed (193.41%) and transected (277.53%) animals 7 days after axotomy (Figure 1C). The intensity of the GFAP immunoreactivity per cell was also elevated in the lesioned facial nuclei (Figure 3A-D). The astrocytic reaction in the facial nuclei induced by the nerve crush or transection was also observed by the increased size of the cytoplasm and processes of the GFAP immunoreactive profiles (Figure 3A-D).

Nerve injuries did not promote changes in the number of NF+CV neurons of the lesioned side of the seven day-axotomized facial nuclei compared to sham rats (1 ± 0.04, 0.91 ± 0.052, 1.08 ± 0.06 of the control, crushed and transected rats, respectively, Figure 1D). Despites of that, the NF immunoreactivity increased in the perykaria, as well as axonal and dendritic fibers of the ipsilateral facial nuclei of both crushed and transected animals (Figure 3E-H).

**Figure 4:**
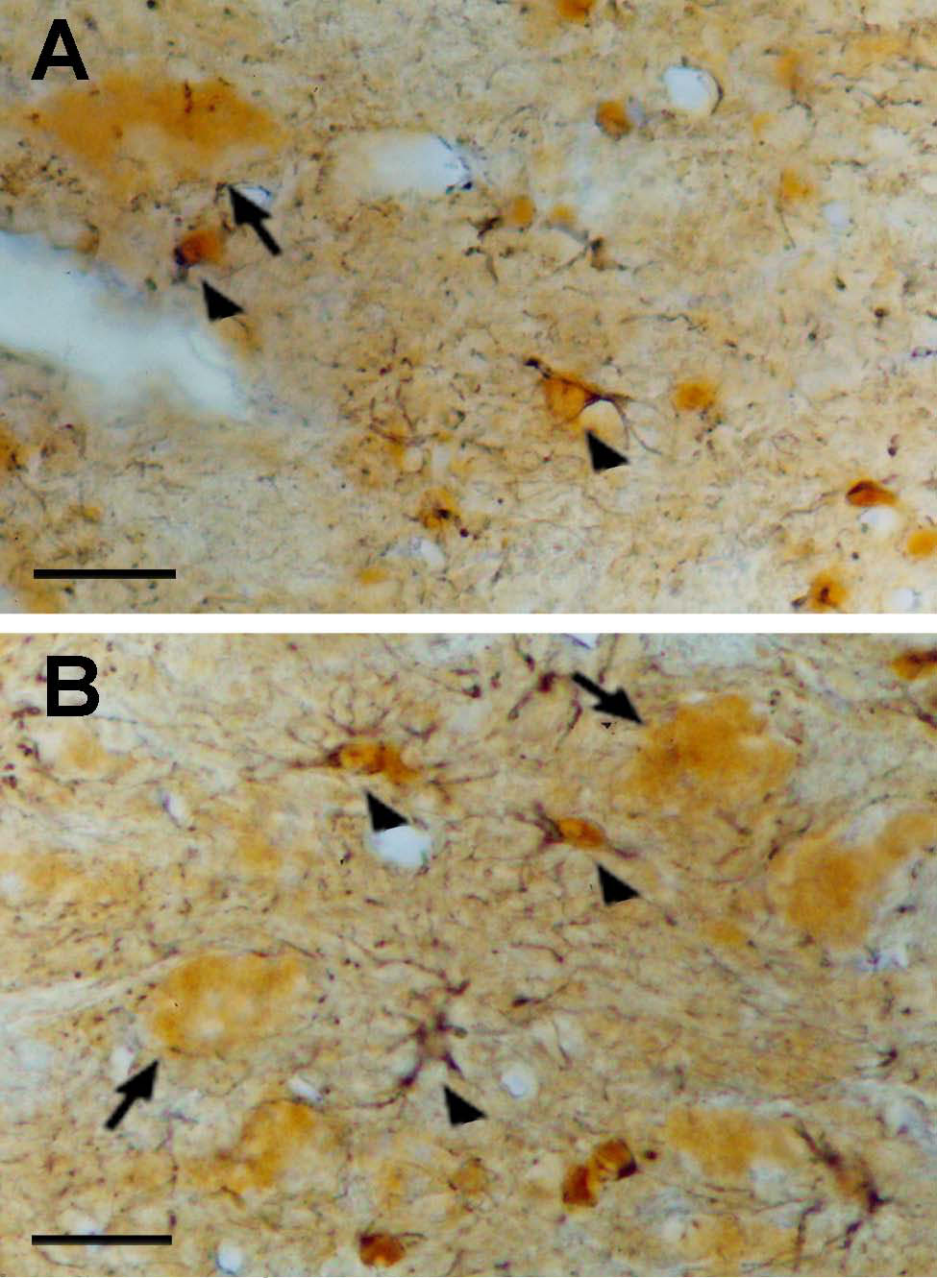
**Color microphotographs showing FGF-2 and GFAP immunoreactivities in coronal sections of rat facial nucleus**. Animals were submitted to the transection of the facial nerve (with amputation of the nerve stumps, **A**, or sham operation, **B**), 7 days before sacrifice. The two-color immunoperoxidase method employing different chromogens was used. The diaminobenzidine (brownish color) and the 4-chloro-naphthol (bluish color) were used for detection of the fibroblast growth factor-2 (FGF-2) and glial fibrillary acidic protein (GFAP) immunoreactivities, respectively. Arrowheads show FGF-2 immunoreactivity in the nuclei of the GFAP immunoreactive astrocytes. It is also seen the FGF-2 immunoreactivity in the cytoplasm of neurons (arrows). Bars = 10 μm.

The two-color immunoperoxidase procedure for the simultaneous detection of the FGF-2 and GFAP immunoreactivities revealed that the vast majority of the nuclear FGF-2 immunoreactive cell profiles were GFAP positive astrocytes in the rat facial nuclei (Figure 4). Furthermore, a higher amount of FGF-2 was found in the nucleus of the reactive astrocytes of axotomized facial nuclei (Figure 4).

The control sections incubated with FGF-2 antibody preadsorbed with human recombinant FGF-2 showed no specific labeling. The control sections incubated with the solvent of the primary and secondary antisera or with the solvent of the avidin-biotin solution showed no immunoreactivity (data not shown).

### FGF-2 in the facial nucleus after systemic corticosterone treatment

**Figure 5:**
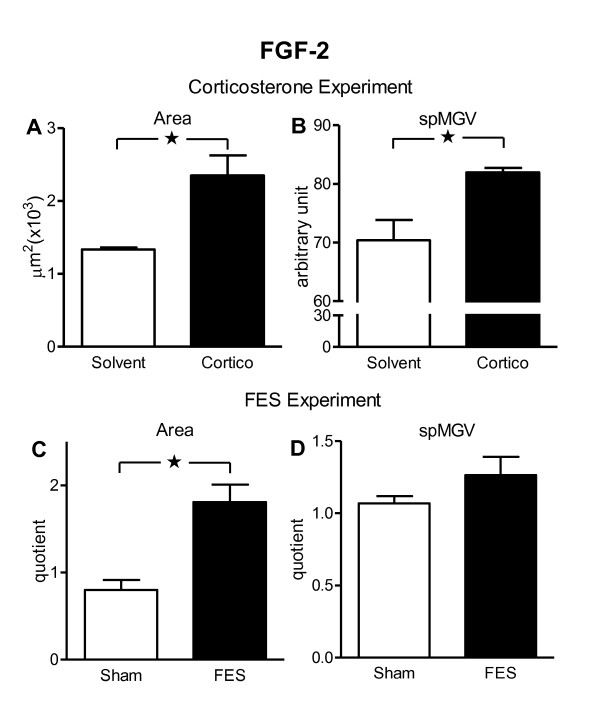
**Effecs of corticosterone or functional electrical stimulation on FGF-2 immunoreactive data of rat facial nuclei**. Figure shows area (**A, C**) and specific mean gray value (spMGV; **B, D**) of FGF-2 immunoreactive profiles in the sampled fields of the rat facial nuclei after systemic corticosterone or solvent injection (**A, B**) and functional electrical stimulation (**C, D**). Measurements were performed in the facial nuclei bilaterally in the corticosterone experiment and ipsilaterally to the levator labii superiors muscle electrode implantation in the functional electrical stimulation experiments. The control animals for functional electrical stimulation received electrode without electrical current. Morphometric/microdensitometric image analysis was used. The measurements represent the FGF-2 immunostaining area (within a 2.56 × 10^-2 ^μm^2 ^sampled field) and intensities (spMGV, arbitrary values) and reflect the number and amount per profile of the measured immunoreactivity, respectively (see text for details). Values are means ± S.E.M.; n = 4-5;*p < 0.05 according to the non-parametric Mann-Whitney *U *test.

As shown in the Figure 5A-B, a seven days-systemic injections of corticosterone resulted in a significant increase of FGF-2 immunoreactivity in the rat facial nuclei as seen from the measurements of FGF-2 immunoreactive area (75.8%) and spMGV (16.4%). The qualitative analysis of the FGF-2 immunoreactivity revealed an increased number of putative glial profiles processing higher amount of the immunoreaction product and only few neurons showing an elevation of the FGF-2 immunoreactivity in the facial nuclei of corticosterone treated rats compared to control animals (illustrated in Figure 2E, F). Procedures for GFAP and FGF-2 double labeling showed the presence of FGF-2 immunoreactivity in the nuclei of astrocytes as demonstrated in the facial nerve injury experiments, however astrocytes have not become reactive after corticosterone treatment (data not shown).

### FGF-2 in the facial nucleus after functional electrical stimulation of the levator labii superioris muscle

A seven days-functional electrical stimulation promoted increases of FGF-2 immunoreactivity in the rat facial nuclei as seen from the measurements of FGF-2 immunoreactive area (127%, quotient of ipsi *vs *contralateral sides) and spMGV (18%, quotient of ipsi *vs *contralateral sides, but without statistical significance) (Figure 5C, D). The qualitative analysis of the FGF-2 immunoreactivity revealed a higher amount of the immunoreaction product mainly in neurons and only few astrocytes showing elevation of the FGF-2 immunoreactivity in the facial nuclei of electrical stimulated rats compared to non stimulated control animals (Figure 2G, H). In this experiment, FGF-2 immunoreactivity was located in the nucleus of astrocytes in the same manner that was found in the other two experiments, however astrocyte have not become reactive after functional electrical stimulation (data not shown).

## Discussion

Retrograde reactions to axotomy leading to morphological and biochemical changes in the neuronal perykaria [35,36] compose a set of responses to maintain the neuronal trophism/plasticity and to trigger axonal regeneration [37-39].

Axotomy of facial nerve applied in this work did not promote changes in the number of NF+Nissl substance stained facial motoneurons either after a crush lesion, which allows immediate fiber growth, or a transection lesion with amputation of nerve stumps. These findings are in agreement and extend previous reports that have demonstrated the resistance of mature motoneurons to axotomy of their fibers [40]. The present findings showing an increased amount of 200 kDa NF immunoreactivity, the major protein of the neuronal cytoskeletal intermediate filament, in the cell bodies and neuropil of axotomized facial neurons are in accordance with previous publications that have demonstrated a remarkable regenerative capacity of motoneurons following axotomy in adult rodents and human beings [41]. Tetzlaff and co-workers [42,43] have demonstrated increased syntheses of the cytoskeletal proteins actin and tubulin after axotomy of the rat facial nerve simultaneously to the enhanced NF contents and a low regulation of NF synthesis. Differential regulation of expression and accumulation of the cytoskeletal proteins in axotomized cell bodies and fibers could be due to their different timing regarding turnover, phosphorilation and participation in specific cell restoration, plasticity and regeneration processes [44].

The retrograde phenomenon following axotomy was also observed by the astrocytic reaction in the injured facial nuclei. Activation of astrocytes has been demonstrated after neuronal lesion [45], electrical stimulation [46], cytokine administration [47] by means of the increases of GFAP immunoreactivity or mRNA. The astrocytic activation has been described to be related to local ionic homeostasis as well as to production of neurotrophic factors [48]. In fact, the paracrine actions leading to neuronal trophic support promoted by the CNS astrocytes have been considered to be important for maintenance and plasticity of the injured neurons [49].

Our findings of increased GFAP immunoreactivity in the facial nucleus following crush or transection lesions of facial nerve are in agreement and extend previous observations which have described retrograde astroglial reactivity after axotomy of cranial motoneurons [15] and also lesions of spinal nerves containing sensory and motor fibers [18,45].

It is well known that the peripheral sensory neurons require a target-derived trophic support [50] and the axotomy of their fibers leads to a partial disappearance of the cell bodies located in the peripheral ganglia [51]. Moreover, axotomy of peripheral motor fibers does not trigger apoptosis of damaged neurons acutely, however a certain degree of a long term cell body atrophy and cell death might take place in the axotomized motoneurons in the cases of regeneration failure [52]. These considerations already underline the importance of autocrine/paracrine mechanisms in the trophic regulation of motoneuron perikarya.

The search for sources of trophic support for peripheral neurons after axotomy has led to descriptions of increased synthesis of neurotrophic factors in the proximal and distal stumps of the injured nerve [53]. Moreover, Heumann and co-workers [54] have observed an increased level of nerve growth factor protein and mRNA in non neuronal cells surrounding the axons of sensory and motor neurons and Levy and co-workers [18] showed increased levels of FGF-2 in the dorsal root ganglia satellite cells surrounding the cell bodies of axotomyzed peripheral sensory neurons. In fact, local production and release of neurotrophic molecules in different parts of compromised neurons may be related to specific functions such as wound repair, trophic support for neuronal maintenance and nerve fiber sprouting/outgrowth [55], this late resembling the reinervation of the distal nerve stump and target [56] when regenerative conditions are offered.

An important finding of the present work was the substantial increases of FGF-2 in the reactive astrocytes of axotomized facial nuclei. It is known that the FGF-2 is a potent survival factor for neurons from different parts of the nervous system and that the molecule can also protect neurons from several types of injury [9,14,57-59]. It is the first time that an upregulation of a neurotrophic factor has been described in the reactive astrocytes close to axotomized facial neuronal cell bodies thus highlighting the importance of paracrine trophic mechanisms to those injured neurons as it has been extensively described for others CNS lesioned regions [60]. Astroglial FGF-2 upregulation in reactive astrocytes of transected facial nucleus was similar to that recently published by our group in the hypoglossal nucleus after injury of their fibers [15]. It is possible that the upregulated astroglial FGF-2 in the axotomized facial nucleus may act as a paracrine factor for cell body maintenance and probably influence axonal regeneration as it has been described for certain types of central neurons[61].

The present study, using a polyclonal antiserum, has also shown the presence of FGF-2 immunoreactivity in the cytoplasm of facial motoneurons, which is in agreement with previous observations that have demonstrated FGF-2 immunoreactivity in neurons of several brainstem nuclei using different polyclonal antibodies [6,8,16,62].

In the present paper we have described moderate elevations of the FGF-2 immunoreactivity in the cytoplasm of neurons without changes in the number of those immunopositive cells following facial nerve axotomy indicating a possible additional autocrine role.

It was reported that the FGF-2 synthesized in the tongue may be retrogradaly transported to the hypoglossal nucleus thus acting as a target derived neurotrophic factor. Actually, a transient down regulation of neuronal FGF-2 immunoreactivity in the ipsilateral axotomized hypoglossal nucleus was described [63], however major events showed by our group have been the upregulation of astroglial FGF-2 in the axotomized hypoglossal nucleus [15] and facial nucleus (this paper).

Indeed, FGF-2 undergoes receptor-mediated internalization and retrograde transport in the central [64] and peripheral nervous system [63]. Because the levels of astroglial reaction (seen by the increased number of GFAP immunepositive cells) and the levels of the changes in the astroglial FGF-2 immunoreactivity were higher after facial nerve transection (without fiber regeneration) than after crush (leading to a favorable regeneration), it is possible that such a regenerative failure-impairing the internalization of FGF-2 synthesized in the periphery might have favored FGF-2 synthesis in reactive astrocytes of transected facial nucleus. Thus, paracrine actions of the astroglial FGF-2 in the facial nucleus might help to maintain the trophism of the facial neurons when fibers are disconnected from the target.

In addition to neuronal lesions [11,13,57,65], different experimental designs have been used to study the role of neurotrophic factors in the CNS. Exogenous administration of growth factors to the brain [9,14,58,66], neuronal stimulation [16], physical activity [67], hormonal manipulation [32,68-70] and electrical stimulation [71] applied in neuronal pathways are also commonly employed.

The ability of exogenous neurotrophic factors to trigger neuroprotection and to prevent diminution of neurotransmitter synthesis following cranial nerve axotomy in the neonatal and adult life has been described. Cuevas and co-workers have shown that acidic fibroblast growth factor topically applied prevents the axotomy-induced neuronal death in the newborn rat facial nerve [72]. Brain derived neurotrophic factor also promoted the survival of the axotomized immature facial motoneurons *in vivo *[73] and attenuated the lesion induced-decrease of choline acetyltransferase (ChAT) immunoreactivity and activity in adult facial motoneurons [74]. Sendtner and co-workers have demonstrated that the vulnerability of motoneurons to axotomy in the early postnatal life is prevented by a local application of cilliary neurotrophic factor (CNTF) [75]. The glial-derived neurotrophic factor was demonstrated to rescue axotomy-induced death of facial neurons and to attenuate the diminution of immunoreactivity in the axotomized facial nucleus of neonates [76]. Finally, implantation of cell lines genetically engineered to release CNTF in the brain of mouse with a progressive neuropathy seems to rescue motoneuron loss [77].

Besides the trophic promoting effects of molecules exogenously administered, the expression of endogenous neurotrophic factors following other types of nerve manipulation gives further evidences of the role of specific molecules for motoneuron survival and regeneration.

Treatment of Bell's palsy is still a matter of controversy and there is a consensus for the need of larger and properly designed clinical trials to evaluate the effects of antiviral drugs, glucocorticoids and other proposed therapies for disease. It has been said that steroids e.g. prednisolone may reduce the nerve swelling-induced damage, leading to a potential recovery in early treatments [3,78].

In fact, the role of hormones in peripheral neuropathology is unknown. It has gained evidence the actions of steroid hormones on nervous system trophism [79] and plasticity [80-82], effects that are probably related to their ability to regulate the expression of neurotrophic factors [70,83-87].

We have shown in this study that systemic corticosterone for 7 days led to upregulation of FGF-2 immunoreactivity mainly in astrocytes of rat facial nucleus. The present findings are in line with our previous observations that adrenocortical steroid administration can increase FGF-2 mainly in the glial cells of the rat substantia nigra [9]. Moreover, dexamethasone, a potent synthetic glucocorticoid agonist, was shown to induce the FGF-2 gene expression in primary culture of rat astrocytes from different CNS regions [86], and also to increase FGF-2 immunoreactivity in the substantia nigra astrocytes [88], further emphasizing the influence of steroid hormones on astroglial FGF-2 mechanisms.

It may be possible that glucocorticoid hormones also modulate astroglial FGF-2 syntheses in the axotomized facial nucleus, as we have described in the model of experimental parkinsonism [32,70], however that was not the matter of the present investigation on non axotomized facial nucleus. Moreover, glucocorticoids might also be able to modulate FGF-2 expression in the neuronal fiber surrounding Schwann cells, which may be potentially involved in sprouting and outgrowth of lesioned axons [18]. Nevertheless, based on the gliogenic, angiogenic and fibroblastogenic actions of FGF-2 and consequently its potential actions on wound repair, glucocorticoid hormones may use FGF-2 signaling on its neurorepair role which is also positive for axonal regeneration [60,89]. We are presently performing experiments on axotomized facial nerve to evaluate further this issue.

Physiotherapy might also contribute to rehabilitation of Bell's palsy. Rather largely employed, the efficacy of acupuncture remains unknown because the available studies do not allow adequate conclusions. Furthermore, neuronal stimulation in general and electrical stimulation in particular seem to improve motor recovery in patients with Bell's palsy [90,91].

Hadlock and co-workers investigated the effects of a local brief electrical stimulation (1 h, 3 V, 20 Hz square wave), a mechanical (manual) target muscle manipulation, or both on functional recovery (whisker movement) of transected and repaired facial nerve of rats [92]. Either therapy alone led to long last better effects than that of untreated rats or animals submitted to an association of the two methods. It seems likely that neuronal activation by afferent inputs triggered by manual stimulation approaches is involved in the functional recovery of the denervated muscles since it is effective in cases of cranial nerve lesions with preservation of the sensory fibers (facial or hypoglossal nerve) but ineffective for the treatment of injury of peripheral nerve containing both sensory and motor fibers [93]. Indeed, manual stimulation was shown to improve function and to reduce polyinnervation without triggering collateral sprouting compared to acute electrical nerve stimulation prior to reconstructive surgery after facial nerve injury in rats [94]. These findings are in line with a recent report that showed failure of whisker functional recovery and collateral axonal branching, and also a reduced motor endplate reinervation after facial nerve repair (end-to-end suture) treated by electrical stimulation in rats [95]. Finally, the nature of electrical stimuli must be consider regarding a potential damage to the nerve tissue as described recently by Sapmaz and co-workers [96] after strong and numerous electrical stimuli to the rat facial nerve ranging from 1 to 5 mA.

All in all, the above considerations seem to be in line with our results regarding the increases of FGF-2 in the facial nuclei of rats submitted to a functional electrical stimulation applied in a facial muscle after local implantation of an electrode.

To our knowledge, functional electrical stimulation has not been applied in rodents, despite some reports have evaluated its efficacy for neurofunctional restoration after facial nerve lesions in rabbits [97-99]. We can not exclude the possibility of retrograde signals may have triggered the increases of FGF-2 in the neuronal cells of facial nucleus after the functional electrical stimulation performed in our works, however it seems possible that proprioceptive reflexes might have exerted an important role in that process.

We should emphasize that based on the results presented in this report, the functional electrical stimulation led to elevation of FGF-2 mainly in the cytoplasm of neurons of facial nucleus, which differed to the findings of systemic corticosterone that promoted elevation of FGF-2 in the nuclei of astrocytes of facial nucleus. Taken together, the present paper opened up new avenues for development and further analyses of therapies for Bell's palsy.

In fact, one possibility is the combination of both strategies: hormonal therapy promoting mainly paracrine actions of glial neurotrophic factors to motoneurons that may be necessary for an acute/subacute thophic support and functional electrical stimulation leading mainly neurotrophic autocrine action that may be necessary for a subacute maintenance.

In line with the above described possibilities, Hetzler and co-workers, by using the rat facial nerve crush, showed positive effects of a combinatorial strategy of electrical stimulation proximal to crush injury site and testosterone propionate (TP) administration (in gonadectomized adult male rats) in enhancing facial nerve regenerative properties. In their experiment, while either single treatment modality of electrical stimulation or exposure to supraphysiologic levels of gonadal steroids has some benefit, such improvements are transitory [100]. The application of both treatment modalities significantly accelerates the functional recovery of multiple functional parameters, with the most important being the time until complete recovery. This significant improvement may be attributed to the ability of each modality to affect different aspects of cellular events associated with axonal regeneration and also to a synergism between the two types of treatment. Whether electrical stimulation affects axonal sprouting in the initial fiber outgrowth phases is a matter that remains to be determined. Whereby electrical stimulation was able to reduce the delay before sprout formation, hormone accelerated the overall regeneration rate, and the combined treatment led to additive effects. Furthermore, the two treatments triggered differential temporal effects on expression of genes related to neurotrophism and neuroplasticity [101] which emphasize a possible importance of associative therapies in modulating specific molecular pathways for neurorestoration of axotomized neurons.

## Conclusion

The presence of the FGF-2 immunoreactivity in the neurons and astrocytes of the facial nucleus indicates that the FGF-2 may be an important growth factor for peripheral motoneurons. Expression of astroglial/neuronal FGF-2 in the facial nucleus may be correlated to local paracrine/autocrine trophic actions to axotomized or stimulated facial motoneurons. The FGF-2 signaling may be explored in the search of new therapeutic target for Bell's palsy.

## Abbreviations

bg: background; CNS: central nervous system; CV: cresyl violet; FGF: Fibroblast growth factor; GFAP: glial fibrillary acidic protein; MGV: mean gray value; NF: Neurofilament; sp:specific

## Competing interests

The authors declare that they have no competing interests.

## Authors' contributions

KFM, CJCSF, AFB, CMS, RTS, GPO performed experimental procedures, surgery, drug administration, electrical stimulation, quantitative analyses and statistics. GC wrote the paper. The authors read and approved the final manuscript.

## Author Details

Department of Neurology, University of São Paulo School of Medicine, University of São Paulo, São Paulo, 01246-903, Brazil.
